# Enhanced Antioxidant and ACE-Inhibitory Activities of L-Theanine Following Formation of the Amadori Compound N-(1-Deoxy-D-Fructose-1-yl)-L-Theanine (Fru-The)

**DOI:** 10.3390/foods15203601

**Published:** 2026-10-10

**Authors:** Fan Li, Jiao Zhou, Minmin Zhang, Guoqiang Zhang, Wenting Sun, Huifang Ge, Renjie Zhou

**Affiliations:** 1 College of Biological and Food Engineering, Anhui Polytechnic University, Wuhu 241000, China; 2240542111@stu.ahpu.edu.cn (F.L.);; 2 National Key Laboratory for Tea Plant Germplasm Innovation and Resource Utilization, Anhui Agricultural University, Hefei 230036, China; 3 Wuhu Food and Drug Inspection Center, Wuhu 241000, China

**Keywords:** Fru-The, theanine, Amadori compounds, antioxidant activity, ACE

## Abstract

Many amino acids, after undergoing the Maillard reaction with reducing sugars to form Amadori compounds, exhibit significantly enhanced functional activity. In this study, the Amadori compounds derived from theanine, N-(1-deoxy-D-fructose-1-yl)-L-theanine (Fru-The), were synthesized by vacuum dehydration method. The structure of Fru-The was characterized along with the evaluation of its antioxidant and Angiotensin-Converting enzyme (ACE) inhibitory activities. The Fru-The was identified by liquid chromatography tandem mass spectrometry. Its major mass spectrometric fragments were [M+H]^+^, [M+H-nH_2_O]^+^ and [AA+H]^+^, among others, which are consistent with those of many Amadori compounds previously verified by our team. And our study demonstrated that Fru-The exhibits greater antioxidant properties (ABTS, FRAP, HRSA) and is significantly superior to theanine. Further studies on ACE inhibition have shown that the IC_50_ value of Fru-Th was 1.400 ± 0.207 mM, whereas theanine showed no obvious ACE inhibition within the tested concentration range up to 10 mM, indicating that Fru-The possesses enhanced ACE inhibitory activities compared to theanine. Fluorescence spectroscopy revealed that the inhibition-related characteristics of ACE by Fru-The may involve both dynamic and static quenching, and the negative ΔG values indicate that the ACE-Fru-The binding process is thermodynamically favorable under the experimental conditions. Specifically, this binding process is endothermic and entropy-increasing (ΔH > 0, ΔS > 0, and ΔG < 0), and it appeared to be more favorable at 37 °C. Molecular docking and circular dichroism spectroscopy further demonstrated that Fru-The binds stably to ACE and significantly alters its higher-order structure. Fru-The exhibits markedly enhanced functional activities compared to theanine and suggest that Fru-The may be a promising candidate for the development of functional tea products.

## 1. Introduction

L-Theanine (The), a tea-specific free amino acid comprising over 50% of the total free amino acids in tea [1]. Over the years, many scholars have discovered that The, in addition to providing the key flavor to tea [2], also possesses a variety of physiological activities that are beneficial to human health, including modulation of neural function [3], alleviation of physical fatigue [4], improvement of cognitive function [5] and reducing high blood pressure [6]. During tea processing, the leaves undergo heating, roasting and drying procedures, free The undergoes a condensation reaction with reducing sugars to form an initial-stage Maillard reaction product—the Amadori compound (N-(1-deoxy-D-fructos-1-yl)-L-theanine, Fru-The), whose presence has been detected in over twenty tea varieties across all six major tea categories (green, white, black, yellow, dark, oolong), with concentrations ranging from approximately 0.02 to 0.4 mg/g [7]. Han et al. [7] found that among the six types of tea, the content of Fru-The in black tea was the highest, ranging from approximately 0.2 to 0.4 mg/g. They believe this phenomenon is caused by the higher degree of fermentation in black tea, which leads to an increase in glucose content.

Amadori compounds (ACs) are the initial products of the Maillard reaction that are widely present in most processed foods, due to the ubiquity of the Maillard reaction in food processing [8]. And although ACs themselves are colorless and odorless, they are an important precursor substance and have a crucial impact on the flavor and color of processed foods [9]. Meanwhile, ACs also have been found to possess many functional activities, including antioxidant [10], anti-inflammatory [11,12], anticancer [13] and antihypertensive [14] activities. Our team has previously demonstrated that several ACs, such as Fru-His and Fru-Gly, exhibit significantly enhanced antioxidant capacity and ACE inhibitory activity compared to their parent amino acids [15,16]. These results demonstrate that the conversion of amino acids into their corresponding ACs via reaction with reducing sugars markedly potentiates their functional activities, which may be attributed to the acquisition of novel chemical properties resulting from molecular structural modification.

L-Theanine is highly abundant in tea leaves, and our previous work has demonstrated that adjusting processing techniques can specifically elevate the contents of ACs in processed foods [17]. Therefore, Fru-The holds great potential for application in the development of highly bioactive tea products. However, most studies have focused on those derived from essential amino acids, while relatively little attention has been paid to the functional activities of ACs generated from non-proteinogenic amino acids with distinctive bioactive properties. Multiple studies have demonstrated that various non-proteinogenic amino acids possessing unique functional activities, such as The and γ-aminobutyric acid, can undergo Maillard reaction to form their corresponding ACs [18,19]. However, whether their functional activities are also enhanced has rarely been reported. In the present study, Fru-The was synthesized via vacuum dehydration and its structure was characterized. Subsequently, Fru-The’s antioxidant and ACE inhibitory activities were compared with The. Furthermore, the molecular interaction of Fru-The with ACE and the ACE inhibition-related characteristics of Fru-The were analyzed using fluorescence spectroscopy, CD spectroscopy and molecular docking. This research offers a new approach to utilize the Maillard reaction to enhance the functional activity of The and develop new functional tea products.

## 2. Materials and Methods

### 2.1. Materials

L-Theanine (The), D-(+)-anhydrous glucose (Glc), FeSO_4_·7H_2_O, trichloroacetic acid, formic acid, potassium ferricyanide, acetonitrile, hydrogen peroxide, methanol, ammonia hydroxide and ferric trichloride were purchased from Sinopharm Chemical Reagent Co., Ltd. (Shanghai, China). Nylon 0.22 μm syringe filters (aqueous) were purchased from Dete Biotechnology Co., Ltd. (Changde, China). Ascorbic acid, 1,10-Phenanthroline and triphenyltetrazolium chloride (TTC) were acquired from Yuanye Bio-Technology Co., Ltd. (Shanghai, China). ABTS (2,2′-azino-bis(3-ethylbenzothiazoline-6-sulfonic acid)) and DPPH (2,2-diphenyl-1-picrylhydrazyl) were obtained commercially from Macklin Biochemical Technology Co., Ltd. (Shanghai, China). Dowex 50WX4 ion-exchange resin, hydrogen form with a mesh size of 200–400, was purchased from Bailingwei Technology Co., Ltd. (Beijing, China). Angiotensin-converting enzyme (ACE, from rabbit lung, EC 3.4.15.1) was obtained from Sigma-Aldrich Reagent (China) Co., Ltd. (Shanghai, China). The angiotensin-converting enzyme assay kit (continuous spectrophotometric method) was purchased from Meikang Biotechnology Co., Ltd. (Shanghai, China).

### 2.2. Synthesis of Fru-The

Referring to the method for synthesizing ACs previously reported by our team [17] with slight modifications. 10 mM The and 20 mM anhydrous glucose were solubilized in 40 mL of ultrapure water, and the reaction conditions for the sample are as follows: 95 °C, 2 h and 100 mbar. The reaction was carried out using a rotary evaporator (R-1001VN; Zhengzhou Greatwall Scientific Industrial and Trade Co., Ltd.; Zhengzhou, China). After the reaction was completed, samples were stored at 4 °C pending further purification.

### 2.3. Purification of Fru-The

Based on the aforementioned method [17], the redissolved Fru-The solution was purified of impurities using the Dowex 50WX4 ion-exchange resin (H^+^). Deionized water was first used to elute about 1 h, then 200 mmol/L ammonium hydroxide was applied to elute the Fru-The fraction. The eluted fractions were subjected to the triphenyltetrazolium chloride (TTC) test and those exhibiting a red coloration were retained. The retained fractions were subsequently analyzed by HPLC-ELSD (Shimadzu, Kyoto, Japan) and collected only those fractions that showed a single peak. These collected samples were initially kept at −18 °C for 20 h and thereafter subjected to lyophilization at −54 °C for 70 h in a lyophilizer (Eco mini; Nanjing Jinshi Instrument & Equipment Co., Ltd.; Nanjing, China). The resulting freeze-dried samples was obtained as a pale yellow powder, which was subsequently subjected to UHPLC-QQQ-MS/MS (1290/6460; Agilent Technologies, Inc.; Santa Clara, CA, USA) analysis for identification.

### 2.4. UPLC-ELSD and UHPLC-QQQ-MS/MS Analysis

UPLC-ELSD Analysis: The Fru-The was analyzed and identified using UPLC-ELSD. The column was an XBridge BEH Amide 5 μm column (4.6 × 250 mm). The mobile phase was formulated with eluent A (acetonitrile) and eluent B (0.1% formic acid solution, *v*/*v*). The column temperature was maintained at 35 °C. Chromatographic conditions: flow rate 1 mL/min, injection volume 20 μL, isocratic elution with 70% A, 30% B and a total run time of 15 min.

UHPLC-QQQ-MS/MS Analysis: Mass spectrometric analysis was performed using an Agilent 1290 ultra-high performance liquid chromatography system coupled with a 6460 triple quadrupole mass spectrometer, with a mobile phase comprising eluent A (0.1% formic acid solution, *v*/*v*) and eluent B (acetonitrile). Chromatographic conditions: column temperature at 35 °C, flow rate at 0.3 mL/min and injection volume at 1 μL; the gradient for mobile phase A was programmed from 95% at 0–2 min, linearly decreased to 90% by 5 min, then to 60% by 7 min, further to 20% by 8 min, followed by a rapid return to 95% at 8.1 min and held at this level until 10 min. The analysis was conducted in ESI^+^ mode with a source temperature of 320 °C, a gas flow rate of 6 L/min, a capillary voltage of 2.5 kV and a collision energy of 15 V. The mass scan range was set at 20–505 *m*/*z*, with a scan time of 100 ms.

### 2.5. Ferric Ion Reducing Antioxidant Power Assay (FRAP)

Following the method of Yu et al. [14] with some adaptations. First, 2.5 mL of phosphate buffer solution (pH 6.6) and 2.5 mL of potassium ferricyanide solution (1%) were sequentially added to 1 mL of Fru-The and theanine samples at different concentrations, followed by thorough mixing, and the mixture was allowed to react in a 50 °C water bath for 20 min. Upon completion of the reaction, 2.5 mL of trichloroacetic acid solution (10%) was added, and the resulting mixture was centrifuged at 3000 rpm for 10 min. Subsequently, 2.5 mL of the supernatant was collected, to which 2.5 mL of deionized water and 0.5 mL of FeCl_3_ solution (0.1%) were added. After the sample was allowed to stand at room temperature for 10 min, the absorbance of the sample at 700 nm was measured using a microplate reader (HBS-ScanX; Nanjing DeTie Laboratory Equipment Co., Ltd.; Nanjing, China). Blank: water replacing sample, processed identically. The reducing power was expressed as the absorbance at 700 nm (A_700_).

### 2.6. Hydroxyl Radical Scavenging Activity Assay (HRSA)

According to the procedure described by Zhou et al. [20], the following were sequentially added to 100 μL of 2.5 mM 1,10-phenanthroline solution: 200 μL of phosphate buffer solution (pH 7.4), 100 μL of the test sample solution at various concentrations, 100 μL of 2.5 mM ferrous sulfate heptahydrate (FeSO_4_·7H_2_O) solution and 100 μL of 20 mM H_2_O_2_ solution. Following thorough mixing and 60 min kept at 37 °C in a thermostatic bath, the reaction mixture was aliquoted (300 μL) into a 96-well plate and the absorbance at 536 nm was monitored with a microplate reader. Blank: water replacing sample, processed identically. The formula is given below:

$$\mathrm{Hydroxyl}\;\mathrm{Radical}\;\mathrm{Scavenging}\;\mathrm{Rate}=\frac{A_{s}-A_{1}}{A_{0}-A_{1}}\times 100\%$$

In this formula, A_S_, A_1_ and A_0_ represent the absorbance values of the sample, the control (containing 1,10-phenanthroline, FeSO_4_·7H_2_O and H_2_O_2_) and the blank (containing only 1,10-phenanthroline and FeSO_4_·7H_2_O), respectively.

### 2.7. ABTS Radical Scavenging Activity Assay

Referring to the method of Xu et al. [21] with slight modifications, 220 μL of pre-prepared ABTS radical solution and 20 μL of Fru-The or The samples at various concentrations were sequentially added to a 96-well plate. Thoroughly mixed and then incubated for 25 min at ambient temperature while protected from light. Upon completion of the reaction, the absorbance was measured at 735 nm using a microplate reader and recorded as A_i_. Blank: water replacing sample, processed identically and the absorbance was recorded as A_0_. The formula is given below:

$$\mathrm{ABTS}\;\mathrm{Free}\;\mathrm{Radical}\;\mathrm{Scavenging}\;\mathrm{Rate}=(1-\frac{A_{i}}{A_{0}})\times 100\%$$

### 2.8. DPPH Radical Scavenging Activity Assay

Referring to the method of J. Yang et al. [22] with slight modifications, 220 μL of pre-prepared DPPH radical solution and 20 μL of Fru-The or The samples at various concentrations were sequentially added to a 96-well plate. Thoroughly mixed and then incubated for 30 min at ambient temperature while protected from light. Upon completion of the reaction, the absorbance was measured at 517 nm using a microplate reader and recorded as A_1_. Blank: water replacing sample, processed identically and the absorbance was recorded as A_0_. Additionally, 220 μL of methanol solution, processed identically and the absorbance was recorded as A_2_. The formula is given below:

$$\mathrm{DPPH}\;\mathrm{Free}\;\mathrm{Radical}\;\mathrm{Scavenging}\;\mathrm{Ability}=(1-\frac{A_{1}-A_{2}}{A_{0}})\times 100\%$$

### 2.9. ACE Inhibitory Activity Assay

Use the automatic biochemical analyzer (Hitachi 3110; Hitachi High-Tech Corporation; Tokyo, Japan) in conjunction with the ACE test kit. The ACE (CAS: 9015-82-1) enzyme from rabbit lungs was adjusted to 50 U/L and mixed evenly with different concentrations of Fru-The or The samples. The reaction was conducted at 37 °C for 20 min. The initial enzyme activity of all samples was 50 U/L. The IC_50_ value is the Fru-The concentration that gives 50% ACE inhibition. The final results were calculated using Graphpad Prism 10.1.2 software.

### 2.10. Fluorescence Spectroscopy Analysis of ACE Inhibition

In order to study the influence of Fru-The on the fluorescence properties of ACE, fluorescence spectroscopy was performed via a microplate reader (Spark; Tecan Austria GmbH; Grödig, Austria). Some adjustments were made based on the method of Zhou et al. [15], the excitation wavelength was set at 230 nm and the emission spectrum was scanned over the range of 280–500 nm. An aliquot of 50 μL of ACE solution with an enzyme activity of 500 U/L was thoroughly mixed with 450 μL of Fru-The solution at various concentrations and incubated at 37 °C for 20 min. After incubation, 300 μL of the mixture was transferred to a solid black 96-well plate for measurement. The experiments were conducted at three detection temperatures of 17 °C, 25 °C and 37 °C, respectively. The Stern-Volmer equation was employed to calculate the quenching constant K_sv_ and the quenching rate constant K_q_.

F_0_/F = 1 + K_sv_[Q] = 1 + K_q_τ_0_[Q]

Here, F_0_ and F as the fluorescence intensities of ACE in the absence and presence of Fru-The, respectively; τ_0_ = 10^−8^ s and use [Q] for the Fru-The concentration.

### 2.11. Analysis of Binding Constant and Thermodynamic Parameters

With reference to Abubakar et al. [23] and Tong et al. [24], the binding constant K_a_ and n can be determined using the double-logarithmic equation. These two parameters are critical for evaluating the binding capacity between ACE and Fru-The and the equation is expressed as follows:

$$\log\frac{F_{0}-F}{F}=\log K_{a}+n\log\left[Q\right]$$

Here, F_0_ and F as the fluorescence intensities of ACE in the absence and presence of Fru-The, respectively; use [Q] for the Fru-The concentration and K_a_ is the binding constant.

The binding interaction between Fru-The and ACE varies with temperature. Therefore, the thermodynamic parameters can be calculated to further elucidate the nature of their interaction. Specifically, the enthalpy change (ΔH) and entropy change (ΔS) can be used to determine the type of interaction forces between ACE and the Fru-The, while the Gibbs free energy change (ΔG) indicates the spontaneity of the reaction. The values of ΔH, ΔS and ΔG were obtained from the following equations:

$$\log K_{a}=-\frac{\Delta H}{2.303\mathrm{RT}}+\frac{\Delta S}{2.303R}$$

ΔG = ΔH − TΔS

where R (gas constant, 8.314 J/(mol·K)) and T (absolute temperature, K).

### 2.12. Circular Dichroism Spectroscopy Analysis

Using a circular dichroism spectrometer (Jasco-1500; JASCO Corporation; Hachioji, Tokyo, Japan), Fru-The and ACE were incubated at 37 °C for 20 min, with the concentration of Fru-The based on the IC_50_ value determined in Section 2.9. CD measurements were performed over a scanning range of 190–400 nm at a scanning speed of 200 nm/min, using a cuvette with a path length of 1 mm.

### 2.13. Molecular Docking Analysis

Fru-The and The were sketched in ChemDraw (V 20.0.0.41) and energy-optimized with ChemBio3D Ultra 14.0, then saved as .PDB files. These optimized structures were subsequently processed in Discovery Studio Visualizer V 23.1.100.23209 (hydrogenation, charge calculation and assignment and rotatable bond setting) and converted to .pdbqt format, while the ACE enzyme (PDB: 1O8A; resolution, 2.0 Å) was acquired from the PDB repository (http://www.rcsb.org/). Chain A was used for docking. Crystallographic water molecules were removed, while the catalytic Zn^2+^ ion was retained because it is essential for ACE activity. Protonation states were assigned at physiological pH 7.0. Ionizable residues of the receptor were protonated using the pKa calculation protocol in Discovery Studio (V 23.1.100.23209), and the dominant ionic forms of the ligands were assigned according to their pKa values. All docking calculations were performed using the CDOCKER protocol (Ligand Interactions module) in Discovery Studio (V 23.1.100.23209), which is a semi-flexible docking approach based on the CHARMm force field. The partial charges of the ligands were assigned using the Momany-Rone method. The docking sphere was centered at x = 42.190, y = 36.698, and z = 47.868 with a radius of 10 Å, covering the ACE active site. Ten poses were generated, and the pose with the lowest CDOCKER interaction energy was retained for further analysis. Non-bonded interactions between the receptor amino acid residues and the docked ligand were visualized and interpreted as predictions of potential interaction modes. The docking and analysis were conducted in Discovery Studio (V 23.1.100.23209) and two-dimensional ligand-protein interaction diagrams were generated to facilitate intuitive observation of the interactions and key amino acid residues involved.

### 2.14. Statistical Analysis

All experiments were performed using three independent batches (*N* = 3). For each batch, every measurement was carried out in triplicate, and the mean value was used for subsequent statistical analysis. Data were organized and plotted using OriginPro 2024 (OriginLab Corporation, Northampton, MA, USA). Statistical analyses were performed using IBM SPSS Statistics version 25.0 (IBM Corp., Armonk, NY, USA). Differences among treatments were assessed by one-way analysis of variance (ANOVA) followed by Tukey’s honestly significant difference (HSD) post hoc test for multiple comparisons. Statistical significance was established at *p* < 0.05. Data are presented as means ± standard deviations (SD).

## 3. Results and Discussion

### 3.1. UPLC-ELSD and UHPLC-QQQ-MS/MS Analysis

**Figure 1:**
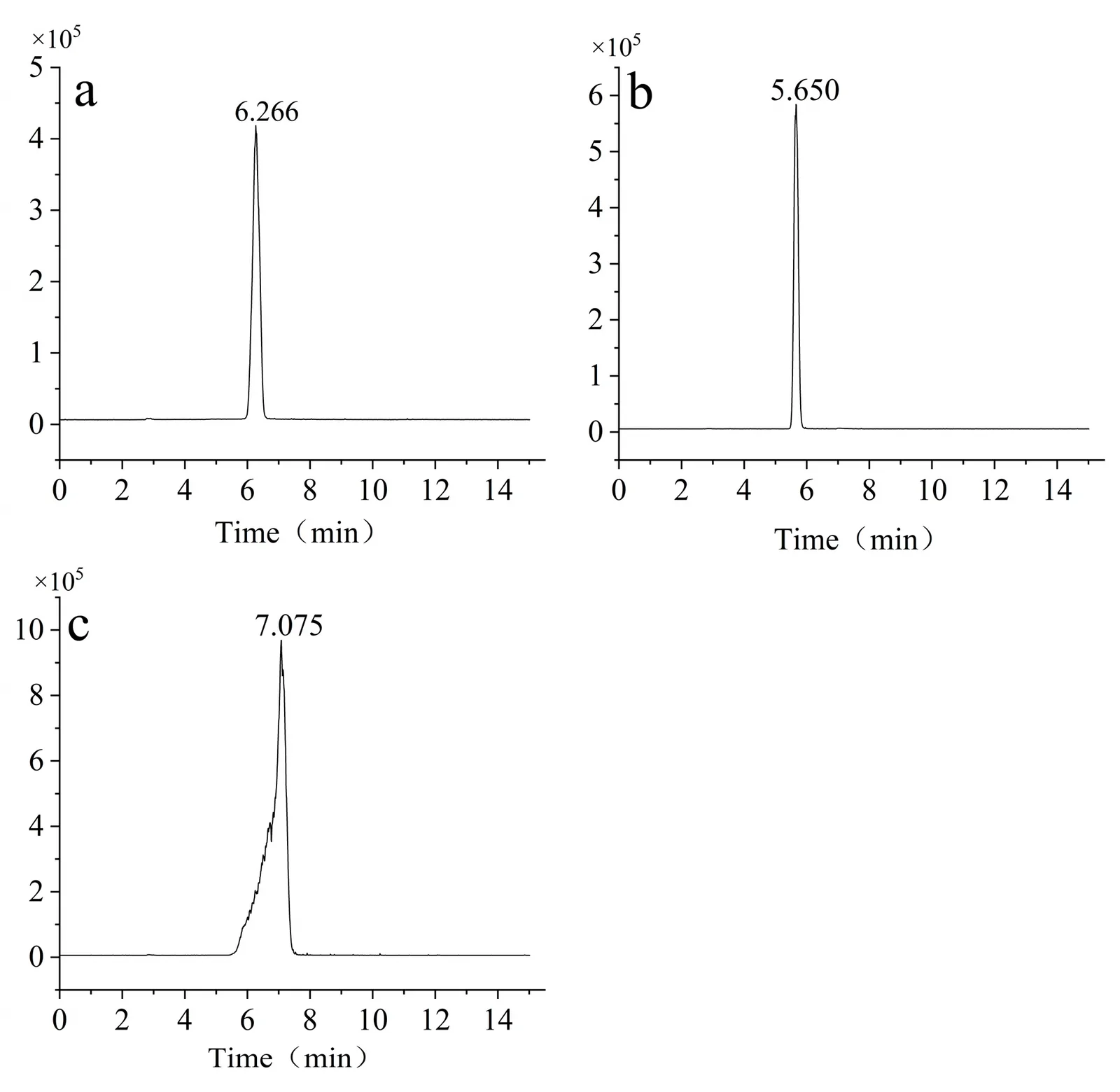
HPLC-ELSD chromatograms of (**a**) glucose, (**b**) theanine, (**c**) and Fru-The.

Our team has previously demonstrated that vacuum dehydration is a more effective method for synthesizing ACs compared to conventional aqueous-phase synthesis [17] and is more suitable for food processing. Therefore, based on the previously established experimental conditions, Fru-The was synthesized via vacuum dehydration and subsequently purified. The HPLC-ELSD chromatogram of the collected fraction is shown in Figure 1. Glucose and The each exhibited a single peak at 6.266 min and 5.650 min, respectively (Figure 1a,b), whereas the samples displayed a peak at 7.075 min (Figure 1c), a retention time that was distinct from those of both glucose and The. This preliminary result indicated that Fru-The in the eluate had been successfully separated.

**Figure 2:**
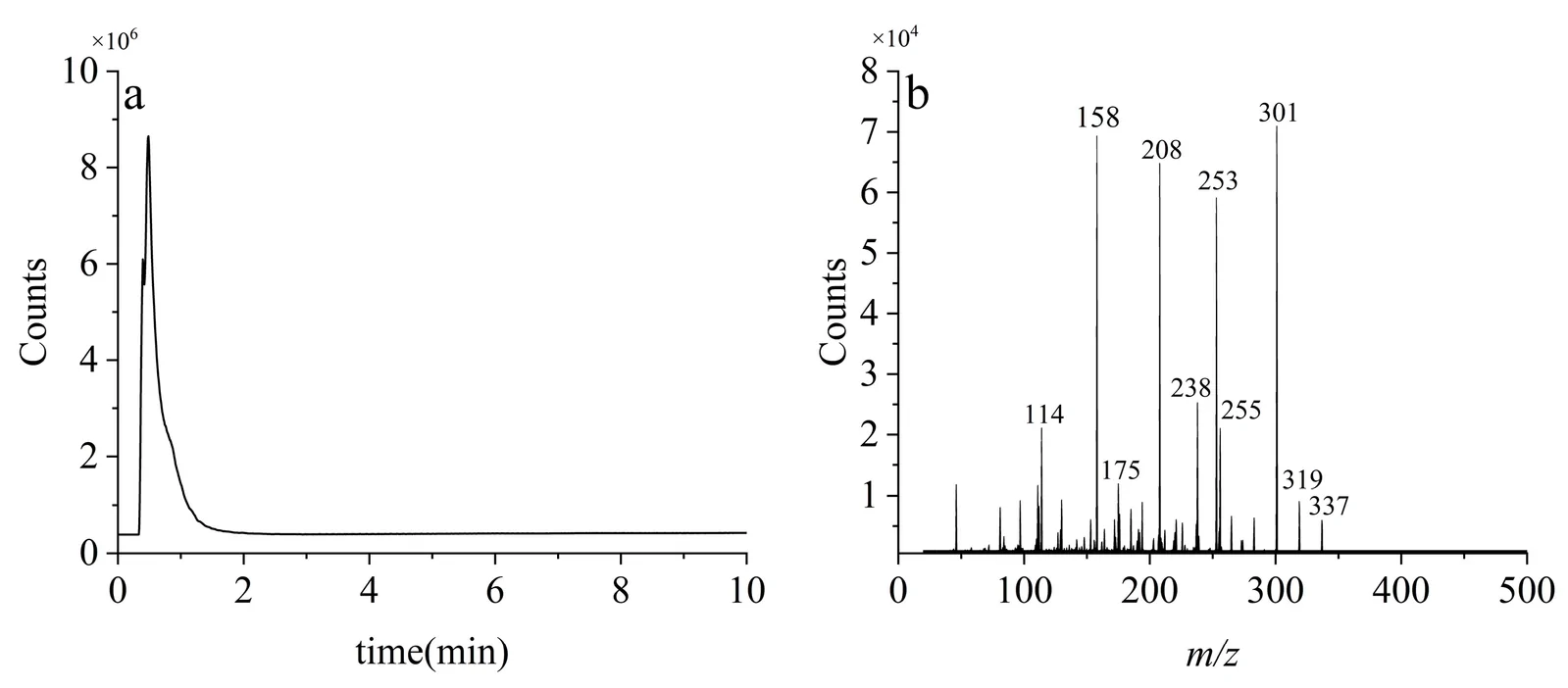
The (**a**) total ion chromatogram and (**b**) mass spectrum of Fru-The.

Fru-The is a short-chain compound that exhibits molecular features of both amino acids and fructose due to the Amadori rearrangement. The mass spectrometric characteristic of Fru-The were identified through UHPLC-QQQ-MS/MS. As shown in Figure 2a, the total ion chromatogram displayed a single peak, consistent with the above HPLC-ELSD results. The mass spectra of the Fru-The is shown in Figure 2b, Fru-The has a molecular weight of 336, and in the ESI^+^ mode, its precursor ion [M+H]^+^ (*m*/*z* 337) undergoes fragmentation due to the removal of water molecules, yielding three fragment ions: [M+H-H_2_O]^+^ (*m*/*z* 319), [M+H-2H_2_O]^+^ (*m*/*z* 301) and [M+H-3H_2_O]^+^ (*m*/*z* 283). Among which the ion at *m*/*z* 301, corresponding to [M+H-2H_2_O]^+^, exhibited a highest response and was identified as the predominant characteristic ion. Additional fragment ions were also observed at *m*/*z* 253 and 208, the ion at *m*/*z* 253 may be formed following partial skeleton cleavage, with subsequent decarboxylation yielding the fragment ion at *m*/*z* 208 [7]. Furthermore, the fragmentation resulted in the cleavage between the amino acid residue and the reducing sugar residue, yielding the The fragment ion at *m*/*z* 175 [AA+H]^+^, which underwent subsequent deamination to produce the fragment ion at *m/z* 158 [AA+H-NH_2_]^+^ [7]. This fragmentation pattern is consistent with the mass spectrometric behavior of other ACs [25,26]. The above results demonstrate that the mass spectrometry patterns of the obtained Fru-The samples are consistent with the theoretical values and those reported in previous literature, and can be applied to the next functional verification experiments.

### 3.2. Analysis of Antioxidant Capacity of Fru-The

After obtaining a sufficient quantity of purified Fru-The, our team first compared the antioxidant activities of Fru-The and The. The antioxidant capacity of Fru-The was evaluated through FRAP, HRSA, ABTS and DPPH assay. In all four assays with The serving as the control, Fru-The consistently demonstrated better antioxidant capacity.

**Figure 3:**
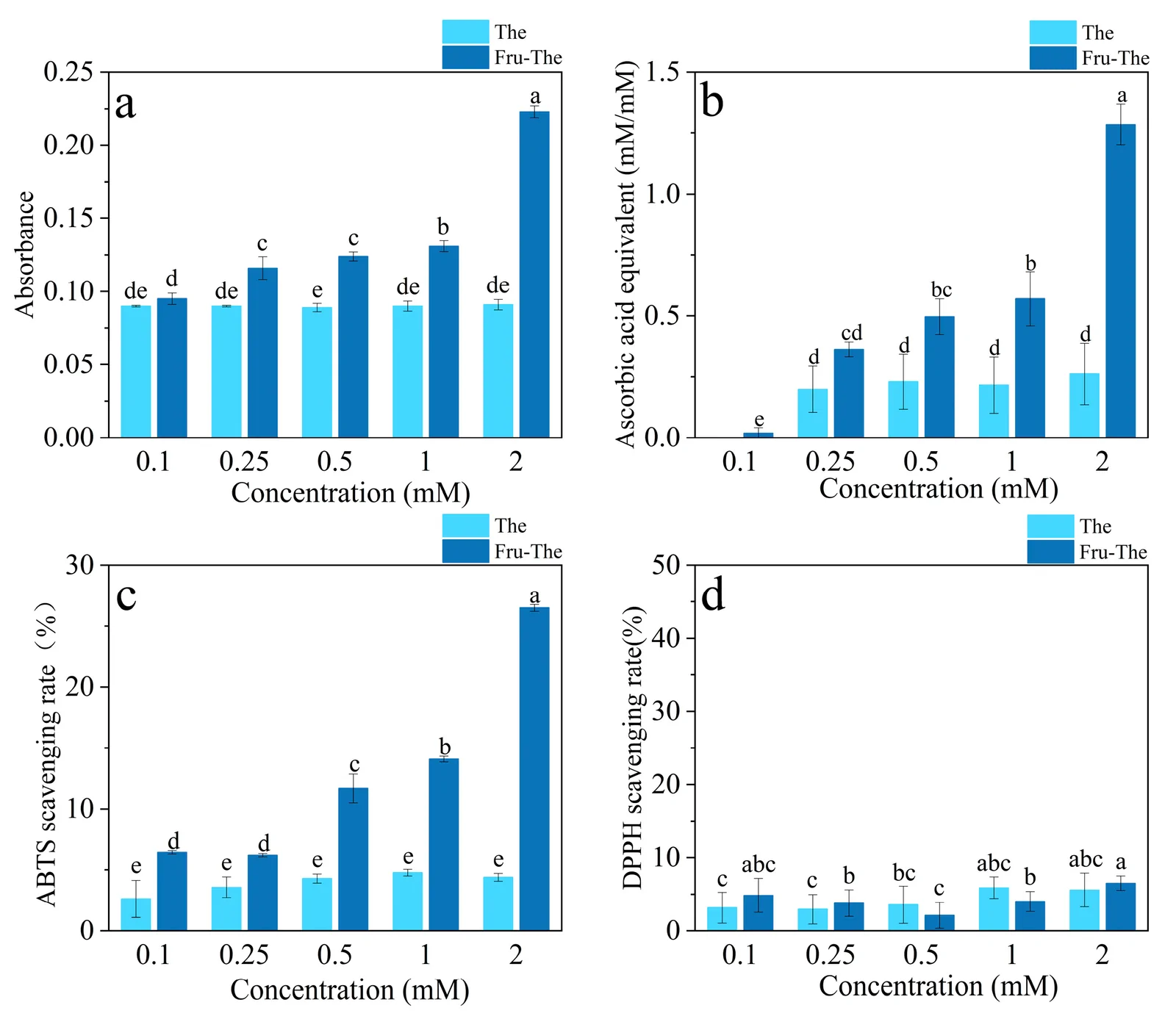
(**a**) Ferric ion reducing antioxidant power, (**b**) hydroxyl radical scavenging activity, (**c**) ABTS radical scavenging activity and (**d**) DPPH radical scavenging activity of Fru-The. Data are presented as mean ± SD (n = 3). Different lowercase letters above the bars indicate significant differences among different concentrations (*p* < 0.05).

As shown in Figure 3a, the FRAP reductive ability was expressed as absorbance intensity, with higher absorbance indicating stronger ferric ion reducing power. L-Theanine exhibited no significant differences with increasing concentration. At the highest concentration of 2 mM, its absorbance was 0.091 ± 0.004. In contrast, Fru-The shows a clear concentration dependence as the concentration increases, with an absorbance of 0.223 ± 0.004 at 2 mM, representing a 2.44-fold enhancement compared to The.

Fe^2+^ ions can undergo the Fenton reaction with H_2_O_2_ to generate Fe^3+^ ions and hydroxyl radicals, thereby markedly elevating the oxidative activity of the system [20]. Fru-The also demonstrated the potential to suppress the Fenton reaction, with an efficacy superior to that of The. As shown in Figure 3b, the HRSA scavenging ability of The at 2 mM was 0.261 ± 0.126 mM/mM (ascorbic acid equivalent, AAE) and no significant variations as the concentration rose (*p* > 0.05). In contrast, Fru-The at the same concentration exhibited a scavenging capacity of 1.285 ± 0.083 mM/mM AAE, indicating a pronounced ability of Fru-The to inhibit the Fenton reaction.

In the ABTS assay (Figure 3c), the ABTS scavenging rate of The did not vary substantially across no significant differences were detected among the different concentrations (*p* > 0.05), whereas Fru-The exhibited a concentration-dependent increase and reached 26.33 ± 0.28% at 2 mM, significantly outperforming the The control (*p* < 0.05).

In the DPPH assay (Figure 3d), Fru-The showed a modest improvement in antioxidant capacity relative to The, but the overall DPPH radical scavenging ability remained at a relatively low level. This may be attributed to the high polarity of Fru-The, which restricts its effective interaction with the hydrophobic and sterically hindered DPPH radical, suggesting that Fru-The contains a greater proportion of hydrophilic antioxidant components [27,28]. Overall, Fru-The exhibited a relatively comprehensive antioxidant capacity across the assays, with most antioxidant effects being significantly stronger than those of The.

### 3.3. ACE Inhibitory Activity Assay

**Figure 4:**
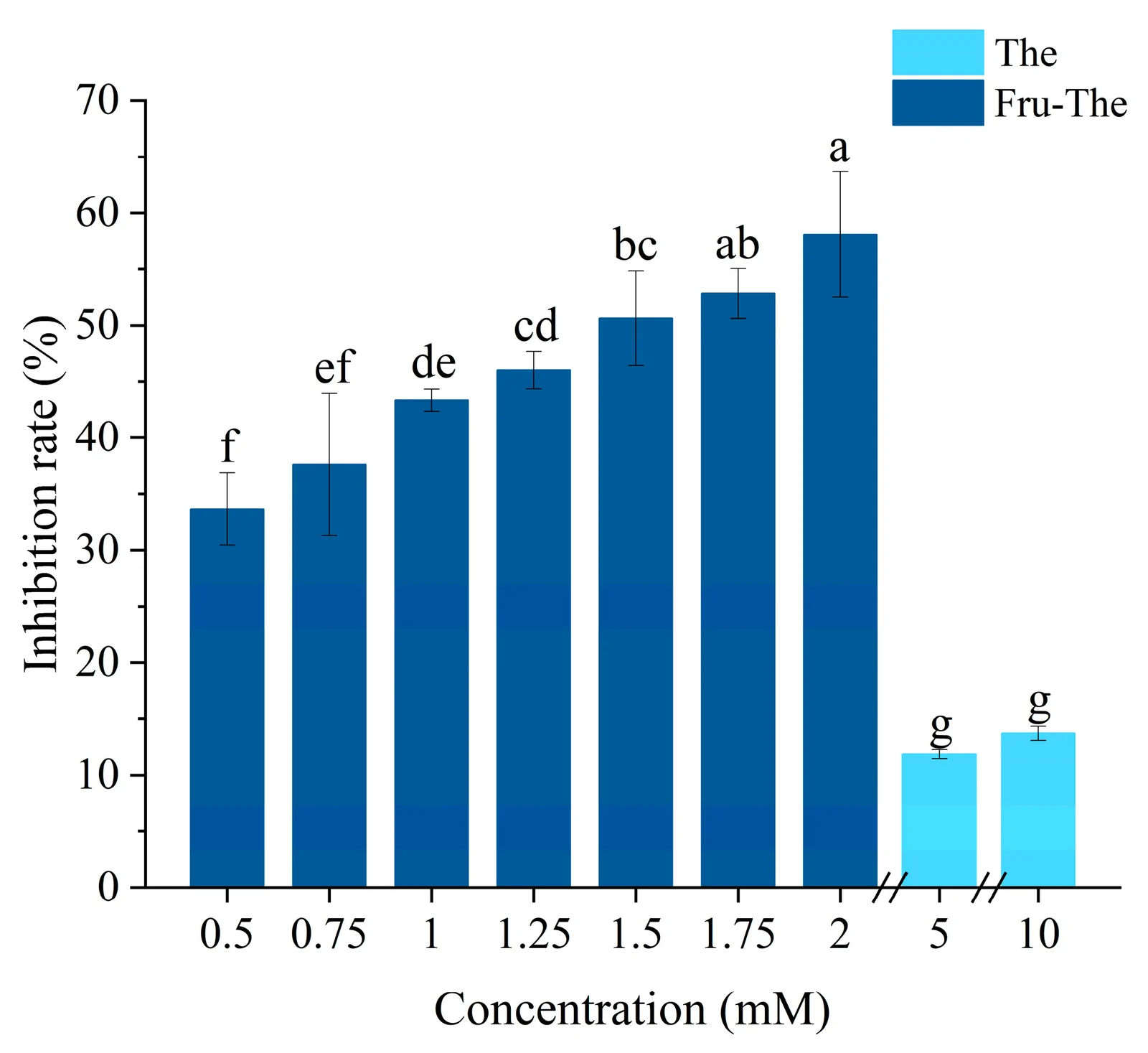
ACE inhibitory activity of The and Fru-The. Data are presented as mean ± SD (n = 3). Different lowercase letters above the bars indicate significant differences among different concentrations (*p* < 0.05).

Angiotensin-converting enzyme is a metalloenzyme that catalyzes the hydrolysis of inactive angiotensin I into the vasopressor angiotensin II, inducing vasoconstriction and elevating blood pressure; thus, blood pressure reduction can be achieved by inhibiting ACE [29]. In this experiment, the effects of Fru-The and The on ACE enzyme activity were investigated using an automatic biochemical analyzer. N-[3-(2-furyl)acryloyl]-L-phenylalanyl-glycyl-glycine (FAPGG) as the substrate, based on the principle that ACE cleaves FAPGG into FAP and GG, resulting in a measurable change in absorbance [30]. ACE with an enzyme activity of 50 U/L was kept with samples of Fru-The and The at different concentrations at 37 °C for 20 min, after which the loss of enzyme activity was determined. As shown in Figure 4, as the concentration of Fru-The increases, its inhibitory effect shows significant differences (*p* > 0.05) and the IC_50_ value of Fru-The was determined to be 1.400 ± 0.207 mM. However, L-Theanine exhibited only 13.41 ± 0.37% ACE inhibition at 10 mM. These results demonstrate that the ACE inhibitory effect of The is relatively poor, but when it transforms into Fru-The, its ACE inhibitory ability will significantly increase (*p* > 0.05). This finding is similar with our previous results [15,16].

### 3.4. Fluorescence Spectroscopy Analysis of ACE Inhibition

**Figure 5:**
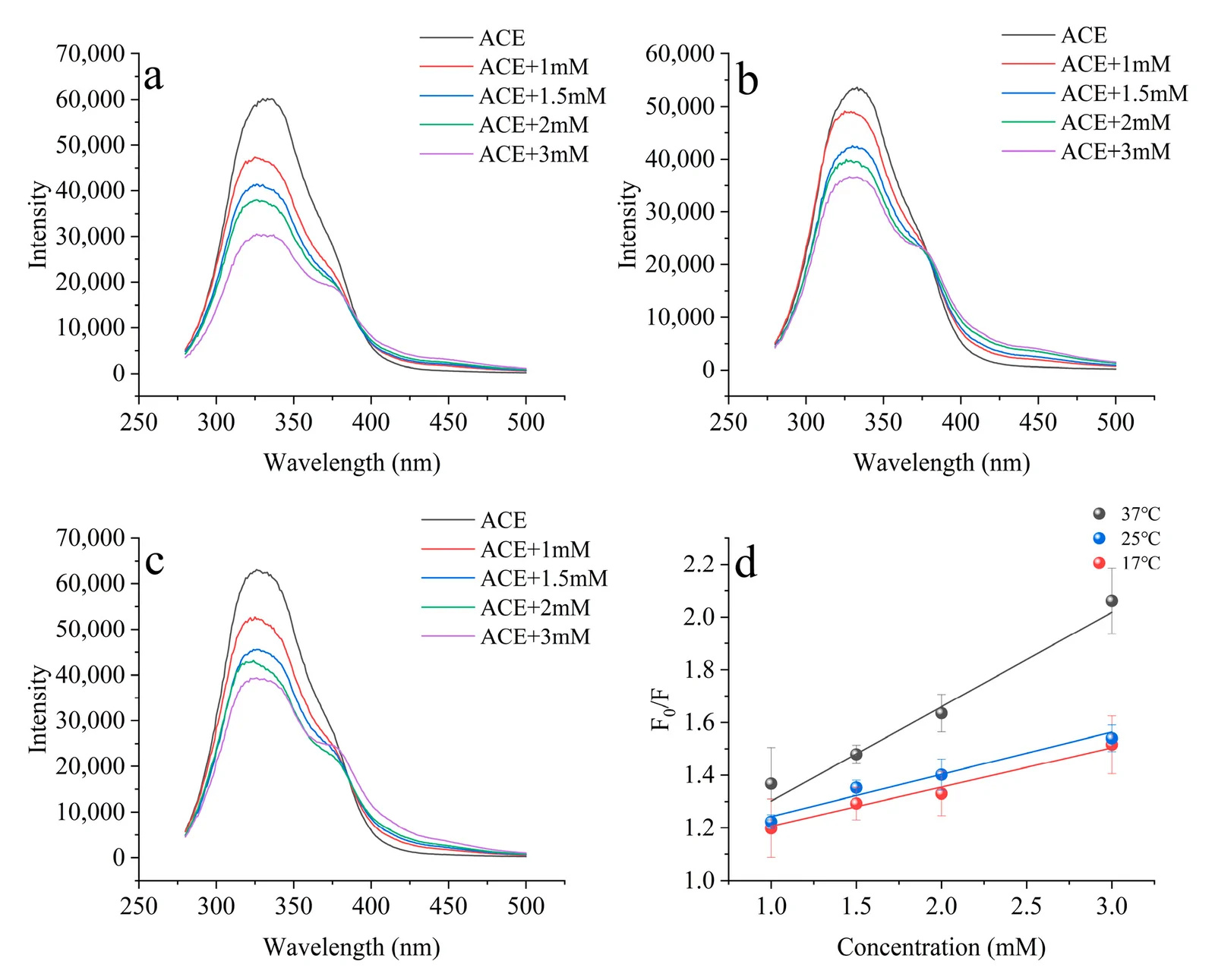
Fluorescence spectra of ACE with Fru-The at (**a**) 37 °C, (**b**) 25 °C, (**c**) 17 °C, and (**d**) F_0_/F plot.

The decrease in fluorescence intensity observed upon the binding of an enzyme to an inhibitor is referred to as fluorescence quenching. Fluorescence spectral analysis can offer essential information concerning the mechanism, mode and affinity constants of inhibitor-enzyme binding. The intrinsic fluorophores of ACE primarily consist of fluorescent amino acid residues within its structure, including tyrosine, tryptophan and phenylalanine [31]. An investigation into the fluorescence spectral alterations of ACE induced by Fru-The was carried out as a function of temperature and the outcomes are presented in Figure 5. The maximum emission wavelengths (λ_max_) of ACE at 37 °C, 25 °C and 17 °C were 333 nm, 330 nm and 326 nm, respectively. A red shift in the λ_max_ of ACE fluorescence was observed with increasing temperature, indicating that ACE is susceptible to environmental temperature changes and that the internal fluorophores migrate toward the external hydrophilic environment as the temperature rises, which is consistent with the findings of Lan et al. [32] and Liang et al. [33]. With increasing Fru-The concentration, a blue shift was observed at all three detection temperatures. At 37 °C, the absorption peak of λ_max_ shifted from 333 nm to 324 nm, suggesting that the fluorophores become more tightly bound and shift toward the internal hydrophobic environment as the inhibitor concentration increases. The mechanism of fluorescence quenching between an enzyme and an inhibitor can be classified as either dynamic quenching or static quenching. The former refers to the collisional interaction between the inhibitor and the fluorophore in the excited state of the enzyme, which reduces fluorescence intensity without altering the enzyme structure; the latter refers to the formation of a non-fluorescent complex between the inhibitor and the enzyme, leading to changes in the enzyme’s microstructure [34]. As shown in Table 1, the K_q_ values exhibited an increasing trend with rising temperature. Notably, the K_q_ value at 37 °C exceeded the threshold of the collisional quenching constant (K_q_ = 2 × 10^10^ L mol^−1^), whereas the values at 17 °C and 25 °C remained below this limit. These results may suggest that the quenching process may shift toward a mixed dynamic and static mechanism at higher temperatures. This observation is broadly consistent with the study by Mahmoudpour et al. [35]. These results indicate that Fru-The can effectively bind to ACE, inhibit its activity and induce a blue shift in the fluorescence spectrum, reflecting a shift toward a hydrophobic environment, with the quenching reaction occurring at 37 °C, 25 °C and 17 °C.

### 3.5. Analysis of Binding Constant and Thermodynamic Parameters

As shown in Table 2, the binding constants (K_a_) of Fru-The with ACE at 17 °C, 25 °C and 37 °C were 0.282 × 10^2^, 0.314 × 10^2^ and 7.791 × 10^2^ L mol^−1^, respectively and the corresponding n values were 0.676, 0.705 and 1.139. A moderate increase in temperature favored the binding of Fru-The to ACE. In conjunction with the thermodynamic analysis presented in Table 3, the binding process was found to be endothermic (ΔH > 0) with ΔS > 0, indicating that elevated temperature promotes the binding interaction, which is in agreement with the maximum K_a_ value observed at 37 °C.

The ΔG values of Fru-The were negative at all tested temperatures, indicating that the binding between Fru-The and ACE proceeded spontaneously at 17 °C, 25 °C and 37 °C. The negative Δ*G* values indicate that the ACE-Fru-The binding process is thermodynamically favorable under the experimental conditions (Table 3). The Δ*G* values appeared to show a trend toward greater favorability at 37 °C, followed by 25 °C and 17 °C. The positive ΔH and ΔS values may indicate that the interaction is predominantly entropy-driven, which could be partly associated with hydrophobic interactions [36,37]. However, other interaction forces cannot be excluded based on thermodynamic data alone. This study observed that Fru-The exhibited a relatively higher binding affinity for ACE at 37 °C, a temperature approaching physiological conditions. This suggests that Fru-The may bind to ACE and exert a certain degree of ACE inhibitory activity at human body temperature.

### 3.6. Circular Dichroism Spectroscopy Analysis

**Figure 6:**
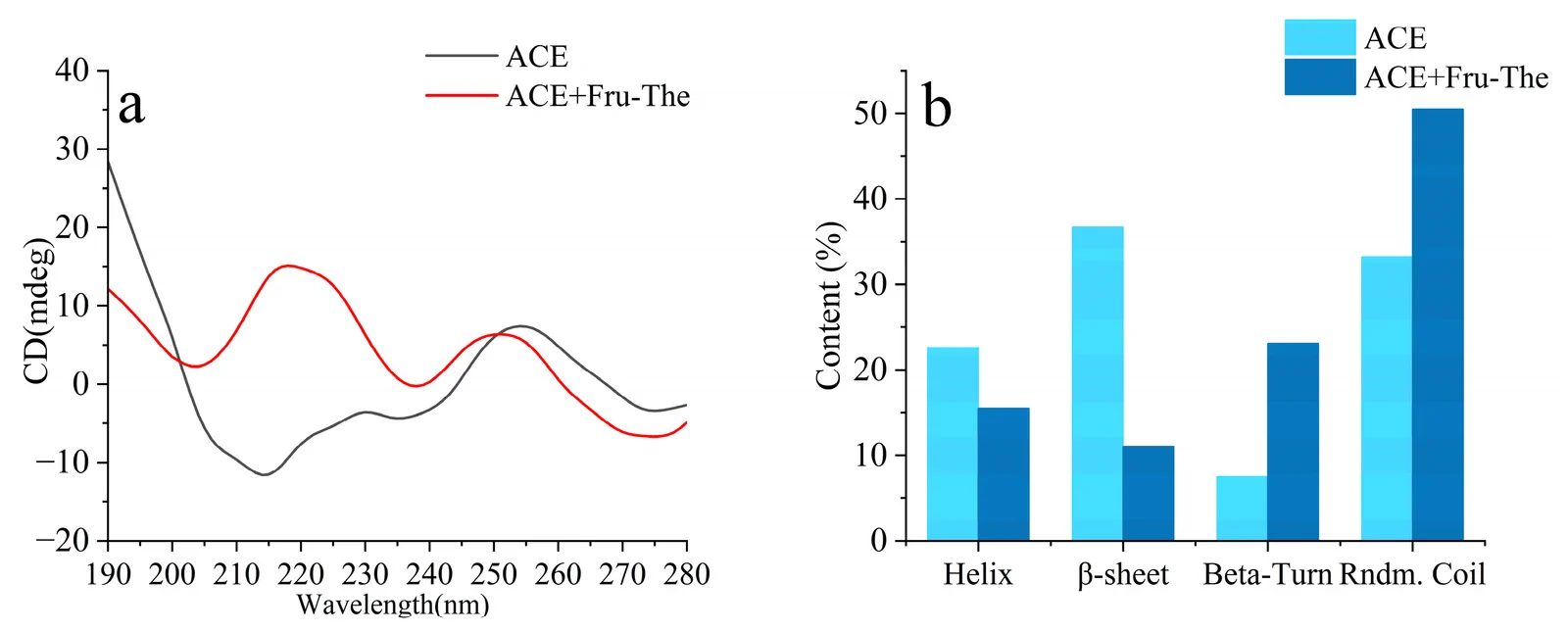
(**a**) CD spectra of ACE before and after interaction with Fru-The and (**b**) the corresponding secondary structure changes.

CD spectroscopy revealed the Fru-The induced secondary structural changes of ACE. As shown in Figure 6, the native ACE protein exhibited a higher-order conformation with coexisting α-helix and β-sheet structures. Upon addition of Fru-The to the enzyme solution, pronounced alterations in the higher-order conformation of ACE were observed. Specifically, the α-helix content decreased substantially from 22.6% to 15.5% and the β-sheet content was markedly disrupted, declining from 36.7% to 11.0%. Concurrently, the β-turn content increased from 7.5% to 23.1% and the random coil proportion climbed from 33.2% to 50.5%, becoming the predominant structural element. These results indicate that the Fru-The exerted a considerable influence on the higher-order structure of ACE, with the ordered structural elements being disrupted and the fractions of β-turn and random coil rising markedly. This may lead to the exposure of new hydrophobic regions, thereby inducing aggregation or degradation, as also demonstrated by Wei et al. [38]. It is speculated that Fru-The can effectively bind to ACE and that their interaction alters the higher-order conformation of the enzyme.

### 3.7. Molecular Docking Analysis

ACE can be classified as a dipeptidyl carboxypeptidase with widespread distribution in somatic and testicular isoforms. Via its HEXXH zinc-binding motif, the enzyme is capable of binding and catalyzing a wide spectrum of substrates and simultaneously interacting with a range of inhibitors. ACE comprises two homologous domains, the N-domain and the C-domain, which serve as the primary active regions of the enzyme. Among these, the C-domain exhibits higher catalytic activity due to the presence of three subsites, S1, S2 and S1′, and represents the core region for ACE binding to substrates such as angiotensin I and bradykinin. This domain also displays dipeptidase activity. Specifically, the S1 subsite is composed of amino acid residues ALA-354, GLU-384 and TYR-523; the S2 subsite consists of residues GLN-281, HIS-353, LYS-511, HIS-513 and TYR-520; and the S1′ subsite is formed by the GLU-162 residue. The substrate catalysis process of ACE can be summarized as follows: hydrogen bonds are formed between the substrate and the active-site residues of the enzyme, while the zinc ion coordinated in the active center serves to activate a water molecule, which then attacks the scissile P1-P1′ carbonyl bond of the substrate, thereby completing substrate hydrolysis [15,16]. In summary, blocking the interaction between the ACE active site, the zinc ion and the substrate represents an effective strategy for inhibiting the enzyme’s activity.

**Figure 7:**
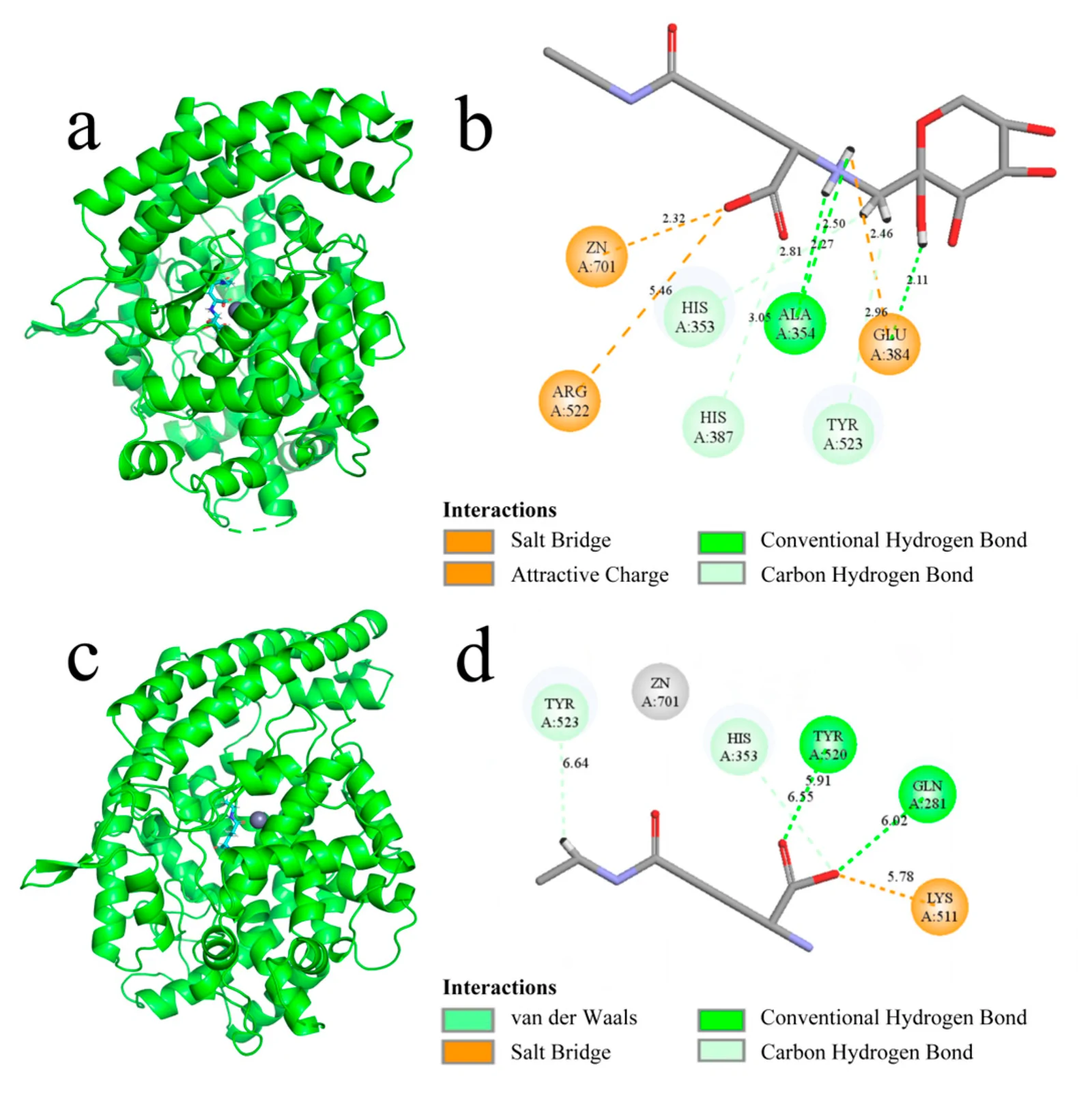
(**a**) Molecular docking simulation of Fru-The with ACE, (**b**) The interaction of Fru-The with ACE amino acid residues, (**c**) molecular docking simulation of The with ACE and (**d**) The interaction of The with ACE amino acid residues.

Molecular docking was conducted using human tACE (PDB ID: 1O8A) as the receptor to simulate the potential interaction between tACE and Fru-The. The lowest-energy conformation of the protein-ligand complex was chosen as the most stable binding mode for further characterization. The docking data are illustrated in Figure 7a,b. The interaction energy (IE) of Fru-The was −65.5542 kcal/mol. The interaction energy (IE) of The was −50.7581 kcal/mol. The more negative predicted IE for Fru-The suggests a potentially more favorable interaction mode of Fru-The with tACE than that predicted for The. The ACE-inhibitory effect of Fru-The is attributable to the establishment of six hydrogen bonds and two salt-bridge contacts with six amino acid residues, as well as electrostatic interactions with the Zn^2+^ ion. Among these, four hydrogen bonds and one salt bridge interaction were formed with four amino acid residues (ALA-354, HIS-353, GLU-384 and TYR-523) located in the S1 and S2 subsites of the ACE active pocket, thereby exerting multi-faceted synergistic inhibition of ACE by occupying the substrate binding site, disrupting the catalytic center and stabilizing the active conformation. In contrast, as shown in Figure 7c,d for The and according to the findings of Grabowski [39], hydrogen bond interaction energy decays exponentially with distance; when the distance exceeds approximately 3.0–3.5 Å, the electron density approaches zero and the orbital overlap and electrostatic stabilization effects essentially vanish. In spite of the hydrogen-bond interactions between The and ACE active-site residues, all hydrogen bond distances were greater than 5.5 Å, exceeding the effective hydrogen bond distance and no interaction with the zinc ion was observed. Collectively, the docking results suggest a potentially more favorable interaction mode of Fru-The with ACE than that predicted for The, which is consistent with the experimental findings.

## 4. Conclusions

This study confirmed that after the formation of Fru-The through the Maillard reaction with The, the antioxidant capacity (FRAP, HRSA and ABTS) and ACE inhibitory activity (IC_50_ = 1.400 ± 0.207 mM) were significantly enhanced. Moreover, Fru-The may bind to ACE via a mixed dynamic and static quenching process and thermodynamic analysis indicates that the interaction may be predominantly entropy-driven. CD analysis revealed that the interaction was accompanied by changes in the secondary structure of ACE. Molecular docking further predicted that the binding mode may involve hydrogen bonds with active site residues and electrostatic interactions with the zinc ion. Collectively, these in vitro findings provide a basis for enhancing functional activities through tea processing and suggest that Fru-The may be a promising candidate for the development of functional tea products. However, further studies, including gastrointestinal digestion, bioavailability, absorption, metabolism, cytotoxicity, in vivo ACE inhibition, blood pressure effects, and stability in food matrices, are needed to fully evaluate its potential for practical application.

## Figures and Tables

**Table 1:** K_sv_ and K_q_ for Fru-The Binding to ACE.

T (°C)	K_sv_ (10^3^ L^−1^ mol)	K_q_ (10^11^L mol^−1^ s^−1^)	R^2^
37	0.353 ± 0.005	0.353 ± 0.005	0.9714
25	0.154 ± 0.020	0.154 ± 0.020	0.9587
17	0.151 ± 0.029	0.151 ± 0.029	0.9473

**Table 2:** K_a_ and n for Fru-The Binding to ACE.

T (°C)	K_a_ (10^2^L mol^−1^)	*n*
37	7.791 ± 1.535	1.139 ± 0.020
25	0.314 ± 0.068	0.705 ± 0.064
17	0.282 ± 0.115	0.676 ± 0.067

**Table 3:** Thermodynamic Parameters of Fru-The Binding to ACE.

Δ*G* (kJ mol^−1^)			Δ*H* (kJ mol^−1^)	Δ*S* (J mol^−1^K^−1^)
37 °C	25 °C	17 °C	131.936 ± 8.257	477.769 ± 25.226
−16.316 ± 0.503	−10.578 ± 0.803	−6.756 ± 1.003		

## Data Availability

The original contributions presented in this study are included in the article. Further inquiries can be directed to the corresponding author.

## Abbreviations

The following abbreviations are used in this manuscript:

Fru-The	N-(1-Deoxy-D-fructos-1-yl)-L-theanine
The	L-Theanine
ACE	Angiotensin-Converting Enzyme
ACs	Amadori compounds
